# Nanotechnology‐Driven Drug Delivery Systems for Lung Cancer: Computational Advances and Clinical Perspectives

**DOI:** 10.1111/1759-7714.70134

**Published:** 2025-07-18

**Authors:** Min Yi, Yiming Li, Hui Jie, Senyi Deng

**Affiliations:** ^1^ Department of Thoracic Surgery and Institute of Thoracic Oncology West China Hospital, Sichuan University Chengdu China

**Keywords:** computational design, drug delivery, lung cancer, molecular simulations, nanotechnology

## Abstract

Lung cancer remains one of the leading causes of cancer‐related deaths worldwide, underscoring the urgent need for transformative therapeutic strategies. Conventional treatments face critical limitations, including poor targeting efficiency, systemic toxicity, and resistance to targeted therapies. Nanotechnology offers promising solutions by enabling enhanced drug stability, bioavailability, and targeting precision. This review integrates recent advancements in nanotechnology‐driven drug delivery systems with a particular focus on computational tools that optimize nanocarrier design. Molecular simulations, quantum mechanics, and AI‐driven models have emerged as powerful approaches to streamline development, accelerate innovation, and enable personalized therapies. Clinically, several nanocarrier‐based formulations have been associated with favorable therapeutic outcomes in lung cancer patients, including extended progression‐free survival and reduced treatment‐related toxicity. Despite these advancements, challenges remain in scaling production, ensuring regulatory compliance, and achieving broad clinical adoption. By addressing these barriers through interdisciplinary collaboration, nanotechnology holds the potential to revolutionize lung cancer therapy and set new standards for precision oncology.

## Graphical abstract

Illustration of nanotechnology‐driven drug delivery systems enabling precision lung cancer therapy through smart carrier design and AI‐powered optimization.

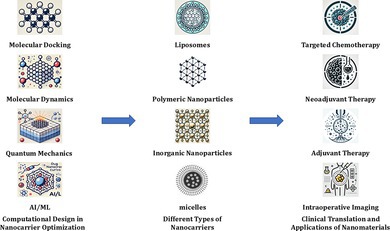

**Abbreviations**
AI: artificial intelligence
EPR: enhanced permeability and retention
FDA: Food and Drug Administration
ICIs: immune checkpoint inhibitors
MD: non‐small cell lung cancer
ML: machine learning
MMP: matrix metalloproteinase
NSCLC: non‐small cell lung cancer
PFS: progression‐free survival
PLGA: poly(lactic‐co‐glycolic acid)
QM/MM: quantum mechanics/molecular mechanics
TME: tumor microenvironment
TME: tumor microenvironment

## 1 Introduction

Lung cancer remains one of the most prevalent and fatal malignancies worldwide, accounting for the highest morbidity and mortality rates among all cancers. According to global statistics in 2020, over 2.2 million new lung cancer cases were reported, with non‐small cell lung cancer (NSCLC) comprising more than 80% of the total [1]. Despite advances in treatment strategies, the 5‐year survival rate for patients with advanced NSCLC remains less than 20% [2]. Moreover, most patients are diagnosed at advanced stages, rendering them ineligible for surgical intervention. Conventional therapies such as chemotherapy and radiotherapy continue to be essential treatment modalities. However, these approaches face several limitations, including poor targeting efficiency, systemic toxicity, and suboptimal therapeutic outcomes [3]. Although chemotherapy and radiotherapy have demonstrated effectiveness in controlling tumor growth, their lack of specificity often results in severe side effects, including bone marrow suppression, gastrointestinal disturbances, and systemic fatigue. For patients with advanced lung cancer, chemotherapeutic agents frequently fail to achieve sufficient concentrations at the tumor site due to rapid drug metabolism and clearance, thereby limiting their therapeutic potential. Similarly, while radiotherapy can provide localized tumor control, it is less effective for patients with extensive metastases and often damages surrounding healthy tissues [4, 5].

Nanotechnology provides innovative solutions to address the limitations of conventional drug delivery methods [6, 7, 8, 9, 10]. In lung cancer therapy, nanocarrier‐based systems have shown improved pharmacokinetics, enhanced tumor accumulation, and reduced off‐target toxicity. Nanotechnology provides innovative solutions to address the limitations of conventional drug delivery methods [10]. In lung cancer therapy, nanocarrier‐based systems have shown improved pharmacokinetics, enhanced tumor accumulation, and reduced off‐target toxicity. Targeted therapies have revolutionized NSCLC treatment by addressing specific genetic abnormalities such as EGFR mutations and ALK rearrangements, resulting in significant extensions of progression‐free survival (PFS) in specific patient groups. However, challenges such as the rapid emergence of drug resistance (e.g., EGFR‐T790M mutation) and tumor heterogeneity limit their long‐term efficacy [11, 12]. Similarly, ICIs, particularly PD‐1/PD‐L1 inhibitors, have emerged as a promising treatment modality for advanced NSCLC. Nevertheless, only 20%–30% of patients benefit from these therapies, and the occurrence of immune‐related adverse events (irAEs) further complicates clinical management [13, 14]. In summary, improving drug delivery efficiency while minimizing systemic toxicity remains a critical bottleneck in lung cancer therapy. Addressing these challenges requires innovative approaches to optimize therapeutic outcomes while reducing side effects, paving the way for more effective and precise treatments.

Nanotechnology provides innovative solutions to address the limitations of conventional drug delivery methods in lung cancer therapy. By enhancing drug stability, bioavailability, and targeting efficiency, nanocarriers have become integral to modern precision medicine. Liposomes encapsulate drugs within phospholipid bilayers, improving solubility and circulatory stability, while reducing systemic toxicity compared to traditional formulations [15, 16]. Polymeric nanoparticles, composed of biodegradable materials such as PLGA, enable sustained drug release by controlling degradation rates, extending drug half‐life, and enhancing therapeutic outcomes [17]. Inorganic nanoparticles, including gold and iron oxide nanoparticles, further broaden nanotechnology's potential by facilitating drug accumulation at tumor sites and offering synergistic effects with other therapies [18, 19]. These advancements highlight the versatility of nanocarriers in overcoming key challenges associated with traditional drug delivery systems, laying a strong foundation for precision lung cancer therapy.

Nanocarriers also enable targeted drug delivery through active and passive mechanisms. Active targeting involves functionalizing nanocarriers with tumor‐specific molecules, such as antibodies, ligands, or peptides, to direct them to tumor tissues. For instance, folate receptor‐modified nanoparticles exhibit superior accumulation in lung cancer tissues, minimizing off‐target effects and reducing systemic toxicity [20]. Passive targeting leverages the enhanced permeability and retention (EPR) effect, wherein the abnormal vasculature of tumors allows nanoparticles to accumulate in the tumor microenvironment (TME). This mechanism enhances therapeutic efficiency even without complex surface modifications [21]. Recent advancements, including cyclic RGD peptide modifications and glycosylation, further enhance nanocarrier specificity, offering new possibilities for precision drug delivery [22].

Intelligent drug delivery systems represent a key advancement in nanotechnology, allowing for tumor‐specific stimuli such as pH, enzymatic activity, or external triggers like heat to control drug release. These systems ensure localized delivery while sparing healthy tissues, significantly enhancing therapeutic precision. For example, enzyme‐responsive nanocarriers utilize tumor‐associated proteases like matrix metalloproteinases (MMPs) to achieve selective drug activation, while photothermal‐responsive nanoparticles combine heat generation with drug release under near‐infrared irradiation, improving tumor ablation and drug penetration [23, 24, 25]. By integrating multiple stimuli, such as pH and temperature, multi‐responsive systems have demonstrated potential in further improving treatment precision and safety [26]. These advanced delivery systems significantly improve therapeutic efficiency while reducing toxicity, paving the way for innovative strategies in precision lung cancer therapy.

Although nanotechnology has revolutionized drug delivery, traditional trial‐and‐error methods for nanocarrier design remain limited by high costs and inefficiencies. These challenges, coupled with the growing complexity of nanocarrier requirements in precision medicine, necessitate advanced computational approaches to streamline development and improve accuracy. Molecular simulation techniques serve as essential tools for optimizing carrier design by predicting interaction patterns and binding energies between drugs and nanocarriers. For instance, molecular docking identifies ligands with high binding affinities, while molecular dynamics (MD) simulations evaluate the stability and behavior of nanocarriers in complex biological environments. Together, these tools accelerate the design‐validation process and improve predictive reliability, significantly reducing development timelines [27].

Artificial intelligence (AI), particularly machine learning (ML), further expands the potential of computational design in nanodrug delivery. ML models leverage vast datasets to predict critical properties of nanomaterials, including toxicity, in vivo distribution, and drug release profiles. For example, deep learning algorithms have successfully estimated the physicochemical properties and biocompatibility of nanocarriers. By integrating multi‐omics data, such as transcriptomics and metabolomics, AI models offer precise insights into nanomaterial–tumor interactions, enabling the design of personalized therapeutic carriers [28, 29]. As computational tools advance, AI‐driven models are expected to enable the rapid creation of next‐generation nanocarriers tailored to individual patient needs. These innovations hold the potential to significantly enhance the efficacy and safety of lung cancer therapies, paving the way for a new era in precision medicine.

**FIGURE 1:**
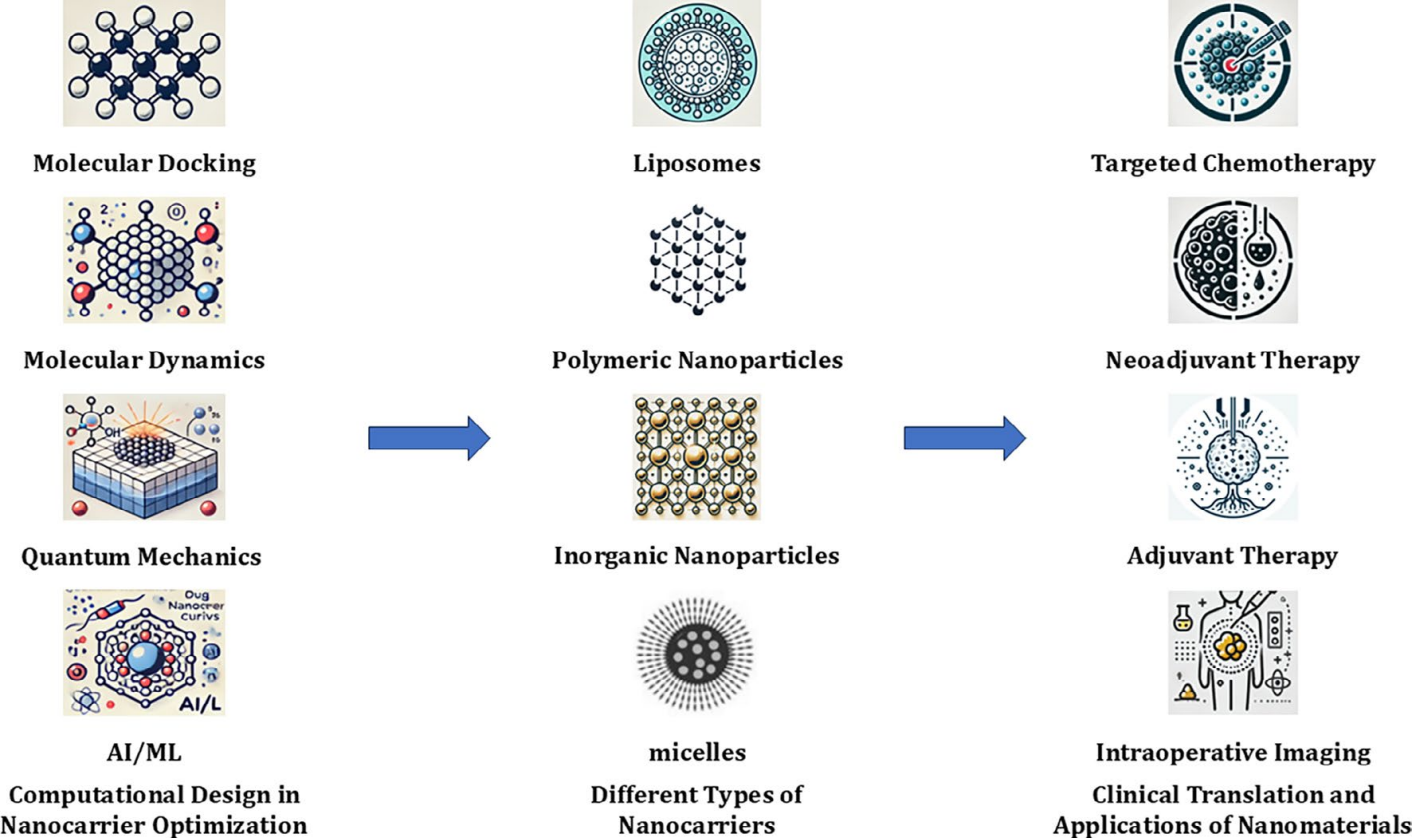
Overview of computationally guided nanocarrier design and clinical translation pathways. Computational tools such as molecular docking, molecular dynamics (MD), quantum mechanical modeling, and AI/ML algorithms inform the rational design of nanocarriers. These platforms—liposomes, polymeric nanoparticles, inorganic nanoparticles, and micelles—are tailored for specific delivery properties. Ultimately, these systems support clinical applications including targeted chemotherapy, neoadjuvant and adjuvant therapy, and intraoperative imaging.

This review aims to systematically summarize key nanocarrier design technologies, with a particular focus on the role of computational design in advancing drug delivery systems. Additionally, it will analyze the application prospects and development trends of nanotechnology in lung cancer treatment, using examples of clinical translation. By integrating theoretical insights and practical applications, this review aspires to provide valuable guidance for precision medicine and personalized therapy (Figure 1). This review is structured to move from computational nanocarrier design methodologies (Section 2) to representative nanoplatforms and formulation strategies (Section 3) and finally to translational challenges, regulatory considerations, and future directions (Sections 4 and 5).

## 2 The Role of Computational Design in Nanocarrier Optimization

The design and optimization of nanocarriers are pivotal to advancing nanotechnology applications in lung cancer therapy. However, the complexity of biological systems and diverse drug delivery requirements make this process challenging. Computational design provides robust theoretical support and technological tools, significantly enhancing design efficiency while reducing research and development costs. This section explores the multifaceted applications of computational design in nanocarrier optimization.

### 2.1 Molecular Docking and MD Simulations

Molecular docking is a widely adopted technique for predicting interaction patterns between drug molecules and nanocarrier surface modification molecules. By calculating binding energies, researchers can efficiently identify the optimal drug–carrier combinations. Tools such as AutoDock Vina, developed by Trott et al. [30, 31], have significantly improved the speed and accuracy of docking calculations, making them widely applicable in optimizing the surface modification of liposomes and polymeric nanoparticles. For instance, studies on paclitaxel‐loaded liposomes revealed that variations in the polyethylene glycol (PEG) chain length of surface modifications markedly influenced drug binding energy and release efficiency, offering foundational insights for targeted delivery strategies [32].

Molecular docking provides fast and efficient prediction of ligand‐binding orientations and affinities, enabling high‐throughput screening of candidate nanocarrier–drug combinations [33]. In contrast, MD simulations explore temporal evolution, structural stability, and drug release behavior in physiological conditions, which are difficult to capture with static models alone [34]. Together, these methods offer a complementary approach to guide both rational design and in vivo performance optimization.

MD simulations complement molecular docking by exploring the dynamic behavior and stability of nanocarriers in complex biological environments. Modern MD tools, such as CHARMM‐GUI and OpenMM, have been extensively employed to simulate the stability of nanocarriers in TMEs, focusing on critical factors like pH and enzymatic activity [35, 36]. For example, MD simulations have demonstrated that drug release rates from liposomes increase significantly in acidic conditions, highlighting the role of hydrogen bonding and electrostatic interactions in governing release kinetics [37]. Similarly, polymeric nanoparticles have shown optimized degradation and drug release profiles in environments rich in MMPs, providing valuable data for designing enzyme‐responsive carriers [38].

In addition, MD simulations have been used to assess the long‐term stability and circulation behavior of inorganic nanoparticles. For example, simulations investigating the behavior of gold nanoparticles under varying pH levels and ionic strengths revealed insights into surface charge density changes, enabling researchers to optimize surface modification strategies. This resulted in enhanced tumor accumulation efficiency while minimizing off‐target toxicity [39]. Coarse‐grained and atomistic MD simulations have revealed pH‐triggered structural disruption in lipid bilayers containing imidazole‐based lipids, supporting their use in acid‐sensitive liposomal drug delivery [40].

### 2.2 Quantum Mechanical Calculations and Nanomaterial Optimization

Quantum mechanics/molecular mechanics (QM/MM) approaches combine the precision of quantum chemistry with the computational efficiency of molecular mechanics, enabling the fine‐tuning of nanocarrier physicochemical properties. Becke's density functional theory (DFT) serves as a robust mathematical framework for calculating intermolecular interactions [41].

For gold nanoparticles, QM/MM has been instrumental in optimizing electronic structures and photothermal properties. By adjusting the electron density distribution of surface‐modified molecules, researchers have significantly enhanced photothermal conversion efficiency—an advancement crucial for gold nanorods in photothermal therapy [42]. Similarly, quantum mechanical calculations for silicon nanoparticles have revealed how carboxyl group distribution influences drug loading capacity, paving the way for improved surface modification strategies [43]. First‐principles simulations combining solid‐state NMR and DFT have successfully resolved interface dynamics in liposil nanocarriers, offering atomic‐level insights into drug release mechanisms from silica‐encapsulated liposomes [44].

### 2.3 AI and ML

AI and ML offer innovative pathways for efficiently designing nanocarriers. By leveraging big data, ML models can rapidly predict the toxicity, in vivo distribution, and drug release characteristics of nanomaterials. For instance, deep learning models have been employed to predict the physicochemical properties and biocompatibility of nanomaterials, providing theoretical support for their biomedical applications [45]. Recent studies further demonstrate that AI algorithms can accurately simulate nanoparticle–tumor interactions, optimize design parameters, and predict therapeutic outcomes in lung cancer models [46]. Additionally, models integrating multi‐omics datasets, such as transcriptomics and metabolomics, can simulate the interactions between nanomaterials and TMEs with high precision, enabling the optimization of drug delivery strategies [47].

Emerging AI technologies, such as generative adversarial networks (GANs), have shown significant promise in designing novel nanomaterials. GANs have been demonstrated to generate nanoparticles with optimized structures, enhancing drug delivery efficiency and targeting performance. This capability allows for the tailored design of nanocarriers to meet specific therapeutic needs [48]. Similarly, support vector machine (SVM) models have been successfully applied to predict nanoparticle distribution patterns in lung cancer models, providing theoretical insights for personalized drug delivery [49].

Beyond these technologies, reinforcement learning (RL) is emerging as a cutting‐edge approach in nanocarrier design. RL algorithms dynamically optimize drug release profiles and material selection, generating nanocarrier designs that perform exceptionally well in complex biological environments [50]. Moreover, graph neural networks (GNNs) are increasingly applied to analyze molecular interactions and structural relationships, predicting more efficient carrier molecules [51]. Table 1 summarizes representative AI models used in nanocarrier design, highlighting their primary applications, strengths, and limitations across lung cancer contexts.

**TABLE 1:** Representative AI models in nanocarrier design.

Model type	Application focus	Strengths	Limitations
CNN	Tumor subtype classification	Strong image/pattern recognition	Requires large labeled datasets
GAN	Nanocarrier structural generation	Creative design, fast optimization	Limited validation in biological systems
SVM	Biodistribution prediction	Effective on small datasets	Less scalable for complex inputs
GNN	Drug–carrier interaction modeling	Captures graph‐based molecular info	Computational complexity
RL	Drug release and formulation	Dynamic optimization capabilities	Requires real‐time feedback modeling

### 2.4 Integration and Clinical Translation of Computational Models

This section bridges the gap between theoretical computational frameworks and their clinical translation, showcasing how integrated modeling approaches contribute to nanomedicine development in real‐world settings. A notable advancement in computational design is the integration of models. By combining molecular docking, MD simulations, and ML, researchers can optimize the entire workflow from nanocarrier design to clinical application. These integrated strategies enable the rapid screening of candidate materials, simulation of drug–nanocarrier interactions, and prediction of in vivo behaviors [52].

Multiscale modeling approaches that link molecular, cellular, and tissue‐level simulations provide a comprehensive perspective on nanocarrier behavior within the biological context [53]. Recent trends have shown promising progress in bridging computational models with clinical nanomedicine. For example, AI‐guided drug release simulations have informed dosing strategies for liposomal formulations in early‐stage clinical trials [54]. Convolutional neural network (CNN)‐based classifiers have been used to predict nanocarrier distribution patterns across tumor subtypes, thereby guiding the selection of optimal nanomaterials for EGFR‐mutant NSCLC patients [55]. Additionally, multiscale modeling integrating molecular simulations with patient‐derived tumor xenografts has been shown to reduce the preclinical evaluation timeline by up to 30%, as reported in recent translational nanomedicine studies [56]. In parallel, GANs have been applied to simulate nanocarrier biodistribution and therapeutic efficacy in EGFR‐mutant NSCLC models, achieving high concordance with patient‐derived tumor imaging data [57]. These advancements further demonstrate the potential of integrated computational frameworks to bridge theoretical modeling with real‐world treatment planning. Figure 1 illustrates this integrated pipeline from in silico design to clinical validation.

Despite the notable benefits, clinical translation of computational designs remains challenging. Validating simulation results biologically is resource‐intensive and time‐consuming. Additionally, many models assume homogeneity within TMEs, overlooking the inherent complexity and heterogeneity of biological systems [58]. Future efforts should focus on developing higher‐accuracy models that integrate experimental validation, improving the predictive power of computational approaches.

Looking forward, advances in computational design tools are expected to further enhance nanocarrier optimization. Innovations such as quantum computing‐based molecular dynamics and multi‐omics‐driven deep learning models are emerging. Furthermore, the integration of computational design with cutting‐edge biomedical technologies—including CRISPR‐based gene editing and immunotherapy—may unlock novel strategies for the treatment of lung cancer. The modeling and optimization strategies described above provide a theoretical foundation for rational nanocarrier development. In the following sections, we examine how these computational principles inform the design of real‐world delivery systems, focusing on structural formulation, drug release behavior, and progress in clinical translation.

## 3 Advancements in Nanocarrier Design for Targeted Lung Cancer Treatment

The application of nanocarriers in lung cancer treatment primarily relies on their ability to improve drug bioavailability, enhance targeting efficiency, and control drug release, overcoming the limitations of traditional treatment methods [59, 60]. In particular, in targeted delivery, nanocarriers, through surface modification or stimulus‐responsive mechanisms, can accurately target tumor regions and reduce drug toxicity to normal tissues. Surface‐modified nanoparticles can enhance the specific binding of carriers to tumor cells, thereby improving treatment efficacy and reducing side effects. Stimuli‐responsive nanocarriers, on the other hand, can intelligently regulate drug release in response to TME features, such as pH and temperature changes, enabling more precise treatment [60, 61]. The optimization of nanocarrier design not only depends on these targeting and response mechanisms but also requires improvement in their biocompatibility, stability, and circulation time in the body [62, 63].

### 3.1 Surface‐Modified Nanoparticles

Surface‐modified nanocarriers significantly enhance targeting and selectivity by binding to specific target receptors. Unlike traditional chemotherapy, surface modification enables nanocarriers to have targeting capabilities [64]. By modifying with antibodies, peptides, or small molecules, nanocarriers can recognize and target tumor cells. These surface‐modified nanocarriers not only significantly increase drug accumulation at tumor sites but also reduce drug toxicity to normal tissues [65]. For example, anti‐EGFR antibody‐modified nanoparticles are widely used in lung cancer therapy. EGFR (epidermal growth factor receptor) is a common receptor on the surface of lung cancer cells, and its overexpression in tumor cells makes anti‐EGFR antibody‐modified nanoparticles an effective targeted therapy strategy [66]. These modified nanocarriers, by binding to the EGFR receptor, enable precise targeting of tumor cells and drug release, improving therapeutic efficacy and overcoming common chemotherapy resistance issues in lung cancer. Similarly, micelles—self‐assembled from amphiphilic molecules—can be modified with targeting ligands (e.g., antibodies or peptides) to enhance their specificity toward lung cancer cells. For instance, a micellar cisplatin prodrug system has demonstrated the ability to simultaneously target and eliminate both lung cancer cells and cancer stem cells, significantly improving therapeutic outcomes [67]. In addition to antibody modification, peptide‐modified nanoparticles have also shown significant results in lung cancer treatment. Peptide‐modified nanoparticles have lower immunogenicity and higher targeting specificity, which allows them to bind to specific receptors on tumor cell surfaces, further enhancing drug uptake and therapeutic effects. Peptide modification not only improves the stability of nanocarriers but also significantly enhances treatment selectivity without triggering strong immune responses [68]. Despite their clinical promise, surface‐modified nanocarriers face challenges such as heterogeneous receptor expression, limited tissue penetration, and variability in endosomal escape efficiency [69, 70, 71]. Comparative in vivo studies with unmodified carriers remain scarce and should be prioritized to guide rational design.

In addition to these approaches, other nanocarrier platforms have also shown promise in lung cancer therapy. Dendrimers—highly branched polymeric nanoparticles—offer precise control over particle size and surface functionality, enabling multivalent drug conjugation and targeted payload delivery [72]. Furthermore, carbon‐based nanomaterials such as carbon nanotubes (CNTs) and graphene oxide exhibit unique optical, electrical, and photothermal properties, facilitating combined diagnostic and therapeutic (theranostic) applications, particularly in nucleic acid delivery and photothermal ablation of lung tumors [73].

### 3.2 Stimuli‐Responsive Nanocarriers

Stimuli‐responsive nanocarriers, which release drugs in response to the TME's characteristics (such as low pH, temperature, and hydrogen peroxide concentration), are a major innovation in nanomedicine. These nanocarriers can trigger drug release according to the specific physiological changes in the tumor tissue, such as acidic environments, temperature variations, and hydrogen peroxide concentration. For example, the pH of tumor regions is generally lower, which provides the theoretical basis for designing pH‐responsive nanocarriers. In acidic environments, these nanocarriers undergo polymer dissociation or conformational changes, releasing drug molecules and thus enhancing the bioavailability of the drugs and improving treatment outcomes [74, 75]. This environment‐sensitive drug release mechanism allows the drug to exert its maximum effect in tumor areas while minimizing toxicity to healthy tissues. Additionally, the high temperature or hydrogen peroxide concentration found in tumor tissues can trigger other drug release mechanisms in stimuli‐responsive nanocarriers. Temperature‐responsive nanocarriers can release drugs in localized tumor regions based on the tumor tissue's higher temperature, significantly increasing the drug concentration in the tumor area and enhancing anti‐tumor effects [76, 77, 78]. Furthermore, hydrogen peroxide‐responsive nanocarriers release drugs according to changes in hydrogen peroxide levels in the tumor region, making them particularly promising in the treatment of drug‐resistant lung cancer [79]. The design of stimuli‐responsive nanocarriers not only improves the targeting and selectivity of drugs but also allows for the automatic adjustment of drug release in response to changes in the TME, greatly enhancing the precision of treatment. Although stimuli‐responsive systems offer enhanced spatiotemporal control, their therapeutic performance may vary depending on tumor microenvironmental conditions such as acidity, enzymatic activity, and oxidative stress levels [80]. More robust preclinical models are needed to predict in vivo efficacy [81].

### 3.3 Carrier‐Free Nanomedicines

Carrier‐free nanoparticle systems, composed entirely of active pharmaceutical ingredients (APIs), have emerged as a promising alternative to traditional nanocarriers [82]. Unlike conventional formulations that rely on excipients or delivery scaffolds, these systems self‐assemble through noncovalent interactions, such as π—π stacking, hydrogen bonding, or electrostatic forces, to form stable nanostructures [83]. These systems exhibit several advantages, including ultra‐high drug loading, prolonged circulation time, and enhanced tumor‐targeting efficiency. For example, peptide–drug conjugate nanoparticles and self‐assembled small molecule nanodrugs demonstrate efficient accumulation in tumor tissues and provide tunable drug release profiles in response to pH or redox conditions [84]. Carrier‐free nanodrugs formed via π—π stacking have shown excellent in vivo stability and the ability to evade immune clearance mechanisms, as demonstrated in recent formulations with optimized physicochemical interfaces [85].

Recent studies also highlight their unique ability to bypass multidrug resistance mechanisms, largely due to their endocytosis‐mediated uptake and lysosomal escape capabilities [86]. In addition, peptide–drug conjugate nanospheres have been reported to overcome efflux transporter–mediated resistance and exhibit high cellular uptake in lung cancer models [87]. Furthermore, certain carrier‐free formulations have demonstrated immunomodulatory effects, including activation of dendritic cells and enhancement of antitumor T‐cell responses, making them suitable for combination with checkpoint inhibitors [88]. Recent evidence has also suggested that some formulations can be designed to co‐deliver immunostimulatory motifs within a carrier‐free framework, potentially augmenting T‐cell priming in the TME [89].

These features position carrier‐free nanomedicines as a next‐generation platform for precision lung cancer therapy. Carrier‐free systems maximize drug loading and minimize immunogenicity, but may suffer from limited structural stability and rapid clearance [82]. Further optimization of pharmacokinetics and combination strategies is essential for successful clinical translation. While these systems show strong promise in preclinical settings, their advancement into clinical use will depend on overcoming challenges related to large‐scale production, safety validation, and regulatory approval—topics addressed in the following section.

## 4 Examples of Clinical Translation and Applications

The clinical translation of nanomedicines in lung cancer has progressed significantly in recent years, supported by advances in drug delivery, targeting precision, and formulation stability. Several nanotechnology‐based therapeutics have entered clinical trials or received regulatory approval for lung cancer and related malignancies.

**FIGURE 2:**
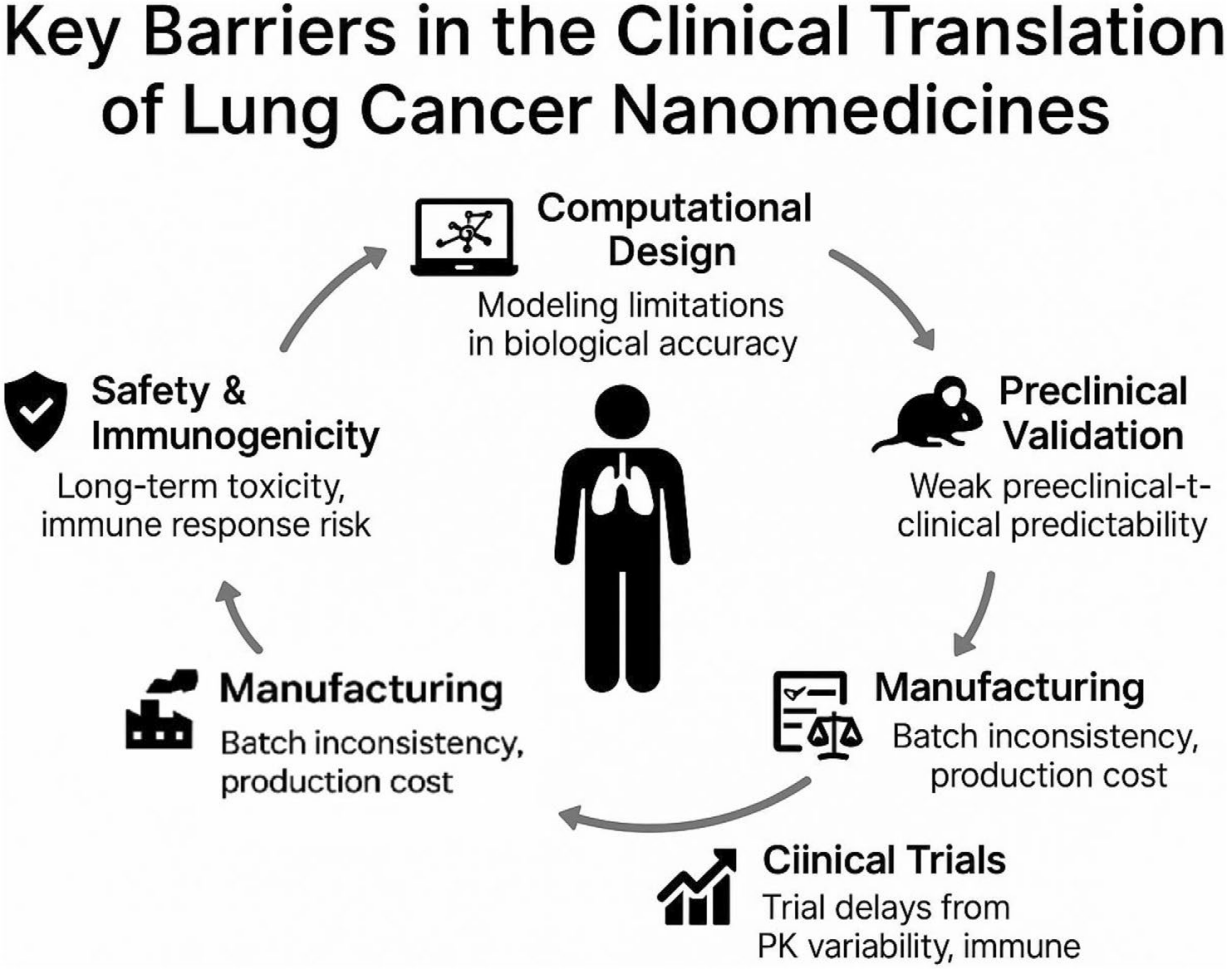
Key barriers in the clinical translation of lung cancer nanomedicines. A visual overview of the major obstacles encountered during the translation of nanocarriers from computational design to clinical application, including issues related to modeling, preclinical validation, manufacturing, safety, and regulatory progression.

To provide a clearer understanding of the key challenges that persist along this path, Figure 2 presents a schematic overview of the major technical and regulatory barriers—from computational modeling to clinical implementation.

### 4.1 Current Status of Clinical Trials of Nanomedicines

The application of nanotechnology has brought transformative advancements to lung cancer treatment by enhancing drug pharmacokinetics and targeting tumor tissues more effectively. Several nanotechnology‐based drugs have progressed into clinical trials or received approval for treating lung cancer and other malignancies.

Abraxane (albumin‐bound paclitaxel) serves as a prime example of nanomedicine. By conjugating paclitaxel with albumin, nanoparticles significantly improve the drug's water solubility and tumor permeability. In a Phase III clinical trial, Abraxane combined with carboplatin demonstrated higher objective response rates (ORR, 33% vs. 25%) and prolonged PFS in patients with advanced NSCLC. Patients also experienced fewer adverse events, such as solvent‐induced allergic reactions and neurotoxicity [90, 91].

Another significant breakthrough is Onivyde (liposomal irinotecan). By encapsulating irinotecan in liposomes, Onivyde prolongs the drug's half‐life and enhances its tumor site concentration. Studies have shown that Onivyde combined with cisplatin exhibits promising antitumor activity in small‐cell lung cancer (SCLC) and other solid tumor models while reducing gastrointestinal toxicity and myelosuppression [92]. Additionally, liposomal systems have been explored for combination immunotherapies. For instance, delivering PD‐1 inhibitors through liposomal carriers significantly enhanced immune responses and improved overall survival (OS) in advanced lung cancer patients [93].

Polymeric nanoparticles are also making significant strides. In preclinical studies on NSCLC models, PLGA‐paclitaxel nanoparticles demonstrated superior tumor accumulation and enhanced stability compared to conventional paclitaxel formulations. These nanoparticles showed reduced systemic toxicity and improved therapeutic tolerance, highlighting their potential as an advanced delivery system for chemotherapeutic agents [94].

Although these nanomedicines showcase promising results, challenges such as dose‐dependent toxicity and patient‐specific efficacy highlight the need for further optimization of nanocarrier designs and personalized treatment strategies.

### 4.2 Preoperative Neoadjuvant and Postoperative Adjuvant Therapy

Nanotechnology offers unique advantages in preoperative neoadjuvant and postoperative adjuvant therapies, which are integral to comprehensive lung cancer treatment.

#### 4.2.1 Preoperative Neoadjuvant Therapy

The primary goal of neoadjuvant therapy is to reduce tumor size, create better conditions for surgery, and improve resection success rates. Nanotechnology enhances drug delivery and accumulation at tumor sites, significantly reducing systemic toxicity and improving treatment tolerance. For example, liposomal doxorubicin (Doxil) has been shown to effectively reduce tumor size in NSCLC models while minimizing drug‐related toxicity. Studies have demonstrated that liposomal doxorubicin increases circulation time and enhances accumulation in tumor tissues, resulting in improved anti‐tumor efficacy and reduced side effects [95]. Additionally, liposomal formulations have shown superior tumor control in preclinical studies [96]. However, due to the limited targeting capability and drug loading capacity of Doxil, its efficacy in certain cancers is insufficient. Additionally, the rapid advancement of immunotherapy and novel targeted therapies has led to a decline in subsequent research on Doxil. Furthermore, its patent expiration and side effects (such as hand–foot syndrome) have reduced the focus on further optimization.

For lung cancer patients with driver gene mutations, such as EGFR or ALK mutations, combining nanotechnology with targeted therapies further improves outcomes. A study on a liposome‐based epidermal growth factor receptor‐tyrosine kinase inhibitor (EGFR‐TKI) delivery system demonstrated significant tumor control and extended survival in animal models, showcasing potential for clinical trials [97]. Another study developed a nanoparticle‐based system that successfully targeted EGFR‐TKI, reducing tumor volume and enhancing treatment efficacy [98].

Moreover, nanomaterials used in photothermal and photodynamic therapies have shown great potential in neoadjuvant settings. A therapeutic platform based on nickel/nickel‐phosphide nanospheres demonstrated remarkable tumor reduction in animal experiments, contributing to tumor downstaging before surgery [99]. Table 2 provides an overview of clinically approved or trial‐stage nanomedicines relevant to lung cancer, focusing on carrier types, delivery mechanisms, and regulatory status.

**TABLE 2:** Nanomedicines in clinical use or trials for lung cancer.

Drug name	Carrier type	Indication	Delivery mechanism
Abraxane	Albumin‐bound nanoparticles	NSCLC (first‐line)	Passive + EPR effect
Onivyde	Liposomal irinotecan	SCLC/NSCLC (in trials)	Passive
Doxil	Liposomal doxorubicin	Off‐label NSCLC	Passive + ligand targeting
BIND‐014	Targeted polymeric nanoparticles	NSCLC (docetaxel‐refractory)	Passive + PSMA targeting
TargomiRs	Bacterial minicells (EnGeneIC)	NSCLC, mesothelioma	miR‐16 mimic + EGFR targeting
Lipoplatin	Liposomal cisplatin	NSCLC	Passive

#### 4.2.2 Postoperative Adjuvant Therapy

The primary goal of postoperative adjuvant therapy is to eliminate microscopic residual lesions, reduce the risk of recurrence, and prolong disease‐free survival. Nanocarriers, with their targeted delivery and controlled release capabilities, have become ideal tools for postoperative adjuvant therapy. For instance, PLGA‐paclitaxel nanoparticles have demonstrated remarkable therapeutic effects in lung cancer recurrence models. These nanoparticles enhance drug circulation time and tumor tissue accumulation, effectively suppressing the proliferation of residual cancer cells and significantly extending survival in animal models. This highlights PLGA‐paclitaxel nanoparticles as a promising postoperative adjuvant therapy strategy to improve treatment efficacy and survival outcomes [100].

Smart‐responsive nanocarriers, such as pH‐responsive liposomes, further enhance the precision and safety of postoperative adjuvant therapies. These nanocarriers can sense the acidic TME and release their encapsulated drugs specifically at the target site, effectively eradicating residual cancer cells while minimizing toxicity to healthy tissues. In animal studies, pH‐responsive liposomes have shown significantly improved drug release efficiency at tumor sites and reduced systemic toxicity, offering a precise and safe tool for postoperative therapy [101].

Furthermore, immunomodulatory nanoparticles have shown great potential in postoperative adjuvant therapy. For example, ICD (immunogenic cell death)‐inducing nanoparticles, carrying tumor‐associated antigens and immune adjuvants, can effectively activate dendritic cells and T cells to mount a robust antitumor immune response, thereby preventing tumor recurrence [102]. In lung cancer models, these nanoparticles significantly enhanced the immune system's ability to recognize and eliminate residual cancer cells without causing severe side effects, showcasing the promising synergy between immunotherapy and nanotechnology [103].

These findings demonstrate that nanotechnology not only effectively suppresses residual cancer cells but also optimizes postoperative therapy through intelligent drug delivery and immune modulation, providing a powerful tool to reduce recurrence rates and improve patient survival.

#### 4.2.3 Integration of Intraoperative Imaging and Delivery

Nanotechnology has also been explored for intraoperative imaging and targeted delivery. Gold nanoparticles doped with fluorescent probes have been utilized to delineate tumor boundaries during surgery, improving resection completeness and minimizing damage to healthy tissues. Similarly, magnetic nanoparticles (e.g., iron oxide) have been applied for real‐time imaging guidance during surgery, combined with drug delivery functionalities for multimodal therapies [104].

For instance, near‐infrared fluorescent gold nanoparticles enabled precise margin visualization and complete tumor resection in murine lung cancer models [105]. Additionally, iron oxide nanoparticles conjugated with doxorubicin demonstrated dual functionality in preclinical studies, facilitating MRI‐based intraoperative tracking while enhancing localized drug accumulation at the tumor site [106].

### 4.3 Drug Delivery Challenges and Regulatory Considerations

Nanotechnology has unlocked remarkable opportunities for drug delivery; however, translating these innovations from the laboratory to clinical applications is fraught with challenges. Several critical issues, including large‐scale production, safety evaluations, and the integration of computational tools, must be addressed to fully realize the potential of nanomedicines.

One significant barrier to clinical translation is the scale‐up production and quality control of nanomedicines. Variability in nanoparticle characteristics, such as size, shape, and surface modifications, can substantially impact drug delivery performance. For instance, inconsistencies between batches of liposomal formulations, including deviations in particle size distribution and drug encapsulation efficiency, can directly affect therapeutic outcomes. Furthermore, ensuring the long‐term stability of nanomedicines during storage and transportation remains a persistent technical challenge [107, 108].

Another pressing concern is the toxicity and safety assessment of nanomedicines. While liposomal carriers are generally well‐tolerated, their metabolic properties and long‐term safety profiles require more extensive evaluation. Inorganic nanoparticles, such as gold nanoparticles, offer promising photothermal therapeutic effects but may induce chronic inflammation or immune system dysregulation due to prolonged retention in the body. Regulatory agencies like the FDA and EMA mandate comprehensive assessments of biodistribution, metabolism, and long‐term toxicity, necessitating rigorous preclinical and clinical studies to support nanomedicine approvals [109, 110].

In addition to general toxicity concerns, specific nanomedicine formulations have been associated with clinically significant adverse events. For example, liposomal doxorubicin (Doxil) has been linked to palmar‐plantar erythrodysesthesia (hand–foot syndrome), a dose‐limiting dermatologic toxicity occurring in up to 50% of treated patients [111, 112]. Albumin‐bound paclitaxel (Abraxane) has been reported to cause sensory neuropathy and hypersensitivity reactions, despite the absence of conventional solvents [113]. Additionally, PEGylated liposomal formulations, such as AmBisome, have been associated with complement activation‐related pseudoallergy (CARPA), characterized by hypersensitivity‐like symptoms due to immune recognition of PEG chains [114, 115]. Certain polymeric nanoparticles, including PLGA‐based carriers, have also demonstrated potential hepatotoxicity or nephrotoxicity in preclinical models, particularly when bioaccumulated or incompletely cleared [116]. These observations underscore the importance of evaluating nanocarrier safety beyond pharmacokinetics, incorporating dermatologic, neurologic, immunologic, hepatic, and renal toxicities as part of comprehensive preclinical and clinical assessments.

In this context, regulatory considerations play an increasingly central role in the clinical translation of nanomedicines. The FDA Nanotechnology Task Force and EMA Reflection Papers outline specific and stringent evaluation criteria, including particle size‐dependent toxicity, sterility testing, and surface chemistry validation [117]. Regulatory case studies further highlight the challenges in this space. For example, BIND‐014, a targeted polymeric nanoparticle encapsulating docetaxel, encountered delays in Phase II clinical trials due to unresolved pharmacokinetic variability between manufacturing batches [118]. Similarly, in the European Union, the approval of liposomal mifamurtide was postponed for over a year pending further immunogenicity assessments [119]. These cases underscore the necessity for harmonized international standards and early consultation with regulatory agencies during development.

Amid these challenges, the incorporation of computational design has emerged as a powerful strategy to streamline the clinical translation of nanomedicines. Techniques such as MD simulations and quantum mechanical calculations provide predictive insights into nanoparticle stability and targeting efficiency, aiding in structural optimization [120]. Meanwhile, AI‐based approaches leverage big data to identify combinations of nanomaterials with high therapeutic efficacy and low toxicity. These computational advancements are enabling the development of personalized treatment regimens while reducing the time and cost associated with drug development [121].

Achieving the successful clinical translation of nanomedicines will require robust interdisciplinary collaboration between materials scientists, biologists, and clinicians. Establishing internationally harmonized standards for quality control and regulatory compliance will further facilitate the approval and commercialization of these advanced therapies. With continuous innovation and a multidisciplinary approach, nanomedicines are poised to revolutionize lung cancer treatment, providing safer and more effective options for patients worldwide.

## 5 Challenges and Future Directions

### 5.1 Technical and Theoretical Challenges

Despite the significant advances brought by nanotechnology in lung cancer therapy, several technical and theoretical limitations hinder its full clinical translation. One of the primary challenges lies in the adaptability and delivery efficiency of nanocarriers in complex biological environments. The TME, characterized by high heterogeneity and dynamic properties such as acidity, enzymatic activity, and vascular permeability, often reduces the accumulation efficiency of nanocarriers at the target site while increasing the risk of off‐target toxicity [122, 123].

Another limitation lies in the unique physiological features of the lungs. The pulmonary environment, with its dense capillary networks, high shear stress, and mucociliary clearance mechanisms, often hinders the efficient deposition and retention of nanocarriers [124]. Additionally, alveolar macrophages rapidly engulf inhaled nanoparticles, reducing their therapeutic concentration at the tumor site [125]. These challenges complicate the application of nanoparticle‐based drug delivery in lung cancer, especially for inhalation‐based formulations.

Additionally, current computational design methods, including MD simulations and AI models, are largely based on static assumptions and fail to capture the dynamic interactions within biological systems, particularly in the tumor immune microenvironment. Moreover, the predictive accuracy of computational models remains limited by incomplete biological data, especially when extrapolating in silico results to clinical scenarios [126]. Most ML models are trained on preclinical or cell line data, which may not reflect the full heterogeneity and complexity of human tumors [127]. Furthermore, current simulation frameworks often lack standardized parameters for nanoparticle–immune system interactions, leading to discrepancies between model predictions and in vivo outcomes. These limitations create a significant translational gap between computational predictions and clinical efficacy, underscoring the need for models incorporating dynamic biological feedback and patient‐derived data sources [128, 129]. At last, the long‐term safety of nanomaterials remains a critical issue. Some inorganic nanoparticles may persist in the body for extended periods, potentially causing chronic inflammation or immune reactions, while the degradation products of biodegradable materials require further toxicity evaluation [130, 131].

### 5.2 Future Research Directions

Future research should focus on integrating advanced technologies with personalized medicine to optimize nanomedicine design and facilitate clinical translation. By incorporating multi‐omics data such as genomics, transcriptomics, and metabolomics, AI‐driven modeling can provide precise simulations of the complex interactions between nanocarriers and TMEs, enabling the development of individualized therapeutic strategies [132]. Additionally, the development of multi‐stimuli‐responsive nanocarriers presents a promising direction. These carriers, capable of responding to multiple signals such as pH, temperature, and enzymatic activity, could significantly enhance drug release efficiency and targeting precision [133, 134].

The combination of nanocarriers with emerging therapies also holds tremendous potential. For instance, nanocarriers designed to deliver immune checkpoint inhibitors (e.g., PD‐1/PD‐L1) or CRISPR‐Cas9 tools can enhance therapeutic efficacy while minimizing off‐target effects [135, 136]. Furthermore, the integration of nanomedicines with cancer vaccines offers new opportunities for lung cancer prevention and treatment, particularly in early intervention and relapse control [137].

From an industrial perspective, the adoption of green manufacturing technologies and sustainable production processes will play a crucial role in the future of nanomedicine. Utilizing biodegradable materials and environmentally friendly synthesis methods can not only lower production costs but also improve biocompatibility and regulatory compliance [138, 139]. Additionally, the establishment of internationally harmonized quality control standards and regulatory frameworks will accelerate the approval process for nanomedicines, promoting their global clinical adoption [140].

### 5.3 Limitations of This Review

This review, although comprehensive, has several limitations. First, the selection of references was limited to published peer‐reviewed articles indexed in major databases and may have excluded relevant unpublished data or ongoing clinical trials. Second, although we attempted to balance discussion across nanocarrier types, clinical stages, and computational tools, some sections inevitably focus more heavily on liposomal and polymeric systems due to their higher representation in current literature. Third, while highlighting promising computational approaches, we did not quantitatively compare model performance or validate predictions against experimental benchmarks. These limitations should be considered when interpreting the generalizability of our conclusions.

## 6 Conclusion

Nanotechnology, combined with computational design, is redefining the future of lung cancer therapy. By enhancing delivery efficiency, reducing toxicity, and improving targeting precision, nanomedicines effectively address many limitations of traditional treatments. This review highlights the critical advancements in nanocarrier design, including their adaptability to complex biological environments, personalized treatment pathways, and sustainable manufacturing practices.

Although challenges remain, ongoing technological innovation and interdisciplinary collaboration are progressively addressing these issues. In the future, international cooperation and standardized regulations will further accelerate the clinical translation of nanomedicines, solidifying their role in lung cancer precision therapy. Through these advancements, nanotechnology promises to provide safer, more effective, and personalized treatment options for patients, marking a significant milestone in the evolution of precision oncology.

## Author Contributions

Min Yi and Yiming Li were responsible for the conceptualization and writing of the article. Zhenyu Yang provided valuable guidance and insights. Hui Jie and Senyi Deng contributed to the guidance and provided financial support for the project.

## Conflicts of Interest

The authors declare no conflicts of interest.

## Data Availability

No datasets were generated or analyzed during the current study.
